# Improved Random Forest Algorithm Based on Decision Paths for Fault Diagnosis of Chemical Process with Incomplete Data

**DOI:** 10.3390/s21206715

**Published:** 2021-10-09

**Authors:** Yuequn Zhang, Lei Luo, Xu Ji, Yiyang Dai

**Affiliations:** Department of Chemical Engineering, Sichuan University, Chengdu 610065, China; zhangyuequn@stu.scu.edu.cn (Y.Z.); dreisteine@stu.scu.edu.cn (L.L.); jixu@scu.edu.cn (X.J.)

**Keywords:** random forest, decision path, fault diagnosis, incomplete data, reliability scores

## Abstract

Fault detection and diagnosis (FDD) has received considerable attention with the advent of big data. Many data-driven FDD procedures have been proposed, but most of them may not be accurate when data missing occurs. Therefore, this paper proposes an improved random forest (RF) based on decision paths, named DPRF, utilizing correction coefficients to compensate for the influence of incomplete data. In this DPRF model, intact training samples are firstly used to grow all the decision trees in the RF. Then, for each test sample that possibly contains missing values, the decision paths and the corresponding nodes importance scores are obtained, so that for each tree in the RF, the reliability score for the sample can be inferred. Thus, the prediction results of each decision tree for the sample will be assigned to certain reliability scores. The final prediction result is obtained according to the majority voting law, combining both the predicting results and the corresponding reliability scores. To prove the feasibility and effectiveness of the proposed method, the Tennessee Eastman (TE) process is tested. Compared with other FDD methods, the proposed DPRF model shows better performance on incomplete data.

## 1. Introduction

There is a large number of variables and parameters in a chemical process, and the complex relationships between them make the chemical process high-dimensional, nonlinear, and strongly coupled. In addition, most chemical processes have long operating cycles, and ensuring that the process is in a normal and safe state of operation while producing qualified products is a challenging issue. With an extended operating time, equipment aging and fouling, and other factors, will lead to a slow decline in the process equipment performance, with the process operation state gradually approaching the security boundary. When a fault occurs, fault detection and diagnosis (FDD) is required to detect and handle the process faults. FDD has always been a hot issue in the field of chemical process safety, and many methods have been proposed and applied [1,2].

Such methods can be divided into the following two categories: model-based methods and data-driven methods [3]. Initially, the FDD approaches were mostly based on mechanism models, using the principle of first nature to model processes and diagnose faults [4,5]. Although these methods are robust and reliable, they are difficult to apply to more complex dynamic processes, due to the high requirements of process experience and knowledge [6]. In contrast, data-driven methods have better applicability to process mechanisms, higher accuracy, and lower requirements for mechanisms, and have become a research hotspot in recent years. Early data-driven FDD methods are mainly qualitative methods, including expert systems (ES), qualitative trend analysis (QTA) methods, and signed directed graphs (SDG), which are difficult to ensure the accuracy of diagnosis, and they cannot efficiently process large amounts of data. Besides, due to the increasing scale and complexity of modern industrial processes, more and more historical data are available, thus quantitative methods based on processing historical data have great advantages in applicability over qualitative methods [7]. Quantitative methods, such as principal component analysis (PCA), independent component algorithm (ICA), and Gaussian mixture model (GMM), are used in FDD. PCA is a powerful tool for dimension reduction, which allows the most important variable information to be retained. It has been extensively used in feature extraction, data compression, image processing, and pattern recognition [8,9]. ICA can process high-intensity, high-noise and related data by extracting independent statistical variables hidden in the process to achieve process dimensionality reduction [10,11,12]. GMM uses different Gaussian components to describe multiple working modes in the general process. The prior probability of each Gaussian component represents the possibility of the process operating under each specific operating condition [13,14]. In recent years, with the popularization of neural networks, scholars have proposed various non-statistical methods; for example, the convolutional neural network (CNN) realizes image recognition and compressed sensor signal processing by automatic feature extraction and deep learning through hidden layers [15,16,17]. The artificial immune system (AIS) [18,19,20] performs well in self-improvement, and can improve the accuracy and efficiency of diagnosis through self-learning in the diagnosis process. These non-statistical methods have become the latest research highlights.

However, although great progress has been made in the theoretical research of the FDD algorithm, and high accuracy has been obtained in toy models, such as the TE process, there are still some difficulties in practical industrial applications. With the rapid development of technologies such as the Internet of Things, and its widespread application in industry, the amount of process data has increased rapidly. Many FDD algorithms may have problems such as low efficiency and an inability to apply the algorithm to multimodal analysis when exposed to massive amounts of data. In addition, due to the frequent mechanical failures or human errors in recording process data, there is often a large number of abnormal or missing values in the data samples collected [21,22], which destroys the continuity of timing data and invalidate statistical methods such as PCA. This brings challenges to the practical industrial application of the FDD algorithm [23]. Therefore, there is a need for a new FDD method that is adapted for handling large amounts of data and is able to handle abnormal or missing data. To handle problems of missing data, four types of approaches are frequently used in the classification and discriminant analysis of incomplete data [24], as follows:

- Deletion of the incomplete feature vectors, and classification of the complete data portion only;
- Development of a multi-classifier corresponding to all the combinations of feature subsets, and classification of incomplete data using the model trained by the same available features;
- Imputation or estimation of missing data, and classification using the edited set;
- Implementation of exploiting procedures for which classification can be still accomplished in the presence of missing variables.

The first method increases bias and may lead to significant information loss [22,25]. The second method can only achieve good results with algorithms such as PCA, when dealing with a very limited number of faults and features, and explodes, in terms of complexity, when numerous features exist in chemical processes [26,27]. The third method uses specific values, such as means and random values, to fill in the missing data, artificially increasing the noise of the data. It is necessary to wait until enough observations are obtained to carry out the imputation, which is time consuming and undermines its effectiveness [28]. For the fourth method, previous studies have shown that some algorithms, such as decision trees, support vector machines, fuzzy algorithms, etc., can effectively process missing values [29,30,31]. Among them, the RF model stands out due to its applicability in the case of missing data [32]. The random forest model is composed of several weak classifiers. Therefore, compared with other statistical methods, it has a strong anti-interference ability. It can operate effectively in large datasets, without dimensionality reduction, and is suitable for high-dimensional input samples [33]. The RF algorithm can also be used in multimodal processes, in which most of the above-mentioned FDD methods are difficult to apply [34,35], and can be classified without feature transformation. In particular, when there is a small amount of missing data in the sample, RF can still maintain good stability [36,37]. However, as the amount of missing data continues to increase, the accuracy of RF will inevitably decrease. Even if the missing values are filled in the way described in the third method above, the subjectivity of the filling method selection and the problem of noise introduction cannot be avoided. Therefore, the question becomes how to use the data information retained in the sample in an objective way to reason and restore the missing values as accurately as possible, so as to alleviate the impact of missing data on the accuracy of the data-driven modelling process, such as FDD. 

Therefore, using the mean values to fill the missing dataset, this paper proposes an improved RF based on decision paths, namely, DPRF, to further modify the results of the RF algorithm to minimize the impact of filling the missing values on the accuracy of the model. According to the importance of each node in the classification process, the algorithm defines a new parameter—reliability score (RS)—to characterize the impact of missing data on the classification, and uses it to correct the original prediction results on the edited dataset to obtain the final classification results. This algorithm will be discussed in detail later in this paper. The main innovations of this paper are listed as follows:

(1). A new FDD algorithm based on random forest is proposed, which is applicable to different missing data patterns.
(2). The concept of decision tree reliability is put forward, based on a decision path to quantify the impact of missing data on the RF model results.
(3). The test performance of the proposed algorithm on the benchmark model is proved to be better than that of other classical FDD algorithms for data loss.

The rest of the paper is arranged as follows. Section 2 reviews the basic mechanism of the random forest classification algorithm and proposes an improved random forest algorithm, defining local importance and reliability scores to modify the prediction results. Section 3 shows how to use the famous Tennessee Eastman process to verify the performance of the improved algorithm. For comparison, the diagnostic properties of traditional RF algorithms and other FDD methods are also tested. Section 4 summarizes this work.

## 2. Materials and Methods

### 2.1. Traditional Random Forest Algorithm

Random forest is a kind of ensemble learning whose core lies in random sample selection and random feature selection. It uses multiple decision trees as the base learner for learning, and applies voting laws to integrate the results of each learner to complete the learning task [38,39].

When the RF model is trained, the structure of the T decision trees is determined, and the optimal characteristics of each split node and the sample size belonging to each category on each node can be obtained. Next, a single sample is input to get the split node that the sample has passed through in a certain decision tree, and the leaf node represents the final classification result of the sample in the decision tree. By arranging these split features in order, the decision path of a specific sample in a specific decision tree can be obtained.

For the features included in the decision path, the amount of information contained in each feature is different, resulting in different importance of each node for target prediction. Node importance is introduced to quantify it.

It is assumed that for a particular sample x, the label predicted by the tth decision tree in the random forest is y. It is defined that the training samples belonging to the y category at the kth node of the decision tree are positive samples. The proportion of positive samples in all training samples contained in this node is denoted as $r_{k}^{t}\left(y\right)$, which can also be considered as the probability that the training sample contained in node k belongs to the predicted sample category y. The difference in proportion of positive samples in child node and its corresponding parent node can be viewed as the node importance of the child node [40,41,42]. The larger the difference, the higher the purity of the sample split to the child node compared to that of the parent node, thus the higher the importance of the child node for the classification problem. Referring to the definition of local increment [42], local importance (LI) of each node for the predicted label can be defined as follows:

(1) $$LI_{k}^{t}\left(y\right)=r_{k}^{t}\left(y\right)-r_{parent\left(t,k\right)}^{t}\left(y\right)$$

where parent(t,k) represents the parent node of the kth node in the decision path of sample x in the tree t.

**Figure 1:**
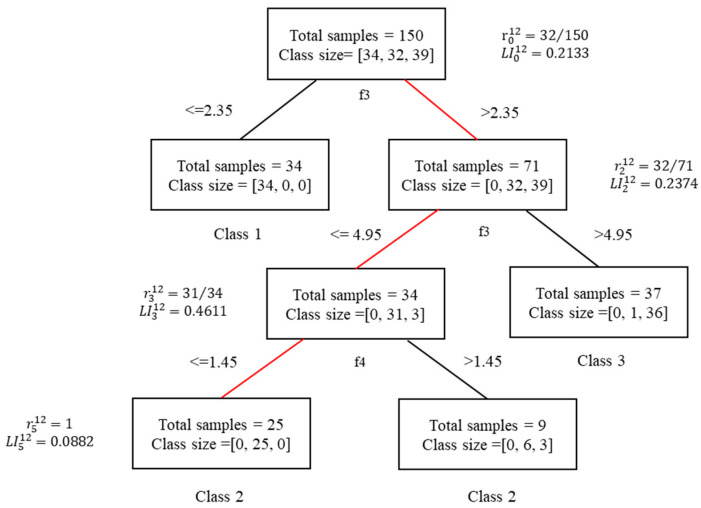
Example of classification of the iris dataset.

More specifically, the iris dataset (https://www.kaggle.com/arshid/iris-flower-dataset, accessed on 14 September 2021) is used as a numerical example to better explain what the decision path is and to give the calculation process of local importance. The iris dataset contains 150 samples, which can be divided into Setosa (class 0), Versicolour (class 1), Virginica (class 2), each containing 50 samples. Each sample contains the following four characteristics: sepal length ($f_{1}$), sepal width ($f_{2}$), petal length ($f_{3}$), and petal width ($f_{4}$). For better training effects, 50 trees are trained and the minimum sample size for each category is set to 5 to prevent overfitting. Take the 12th tree in the RF model for example, whose internal structure is shown in Figure 1 below. Class size on each node represents the number of training samples belonging to each category; for example, class size at the root node equals [32,34,39], which means that 34 samples belong to class 0, 32 samples belong to class 1, and 39 samples belong to class 2. At the root node, $f_{3}$ is the optimal split feature based on the Gini coefficient, and if the value of the third feature of the input sample is no more than 2.35, it moves to the left node. Otherwise, it will move to the right node. Thus, all input samples are divided into two categories. Similarly, the left child nodes and right child nodes then select the optimal split characteristics to split collectively, and the split process will continue repeatedly until the classification pruning requirements with a minimum sample number of 5 are reached. The specific information for the test sample $x_{6}$ is shown in Table 1.

$x_{6}$ satisfies the following:

$$f_{3}=3.9>2.35,\;f_{3}=3.9\leq 4.95,\;f_{4}=1.1\leq 1.45$$

Therefore, the decision path will be recorded as $n_{0}\to n_{2}\to n_{3}\to n_{5}$ as shown in the red path in Figure 1. The corresponding decision nodes set is denoted as $𝒟𝒫=\{n_{0},\;n_{2},\;n_{3},\;n_{5}\}$. The final predicted sample category of sample $x_{6}$ is 2, and the local importance of each node in the decision path of the sample $x_{6}$ can be calculated according to Equation (1).

(2) $$n_{0}:LI_{0}^{12}\left(y=2\right)=r_{0}^{12}=\frac{32}{150}=0.2133\;n_{2}:LI_{2}^{12}\left(y=2\right)=r_{2}^{12}-r_{0}^{12}=\frac{32}{71}-\frac{32}{150}=0.2374\;n_{3}:LI_{3}^{12}\left(y=2\right)=r_{3}^{12}-r_{2}^{12}=\frac{31}{34}-\frac{32}{71}=0.4611\;n_{5}:LI_{5}^{12}\left(y=2\right)=r_{5}^{12}-r_{3}^{12}=\frac{25}{25}-\frac{31}{34}=0.0882$$

where $LI_{k}^{t}\left(y=2\right)$ represents the local importance of the node k on the decision path of $x_{6}$ in the decision tree t with predicted label y=2. All the results are shown in Figure 1 where $LI_{3}^{12}>LI_{2}^{12}>LI_{0}^{12}>LI_{5}^{12}$ indicates that in the classification process of sample $x_{6}$ in the 12th decision tree, node three is the most important with the largest increase in sample purity compared to the parent node. Node two has the second highest importance, and node five has the worst importance. It can be inferred that the data missing on node three will lead to the most significant error in the prediction result.

The corresponding classification result can be obtained on each decision tree similarly to the above procedure for each sample. The idea of the ensemble algorithm is to obtain the final classification results from the classification results of all weak sub-classifiers according to certain voting rules. In the RF model, there are the following two voting rules: soft voting rule and hard voting rule. A brief introduction is given.

Hard voting

Under the hard voting rule, each decision tree can give the classification result of a specific sample. According to the majority voting law, the result with the most occurrences is the final classification result. Its expression is as follows:

(3) $$H_{\mathrm{RF},\mathrm{hard}}\left(x\right)=\underset{y\in Y}{\mathrm{argmax}}\;\left\{\overset{T}{\underset{t=1}{\sum }}v_{t}\left(x,y\right)\right\}$$

(4) $$v_{t}\left(x,y\right)=\{\begin{array}{c} 1,f_{t}\left(x\right)=y\\ 0,f_{t}\left(x\right)\neq y \end{array}$$

where x represents the test sample. Label value y belongs to Y, and Y represents a collection of all possible classification results. $f_{t}\left(x\right)$ represents the classification result of decision tree t for x. $v_{t}\left(x,y\right)$ is the voting variable and $H_{\mathrm{RF},\mathrm{hard}}\left(x\right)$ represents the final classification results based on the hard voting. 

Soft voting

The average value of the probabilities of all sub-classifiers predicting samples to be a certain category is used as the deciding criterion, and the category with the highest probability is selected as the final classification result. Its expression is as follows:

(5) $$H_{\mathrm{RF},\mathrm{soft}}\left(x\right)=\underset{y\in Y}{\mathrm{argmax}}\;\left\{\frac{1}{T}\overset{T}{\underset{t=1}{\sum }}P_{t}\left(x,y\right)\right\}\;$$

where $P_{t}\left(x,y\right)$ represents the probability of the prediction label belonging to y in the decision tree t.

### 2.2. Improved RF Algorithm Based on Decision Path

The traditional RF classification algorithm uses decision trees such as CART as the base learner and applies a certain voting rule to summarize the results of all base learners, so RF has good stability. In this way, even if there is a small amount of missing data in the sample, it will only affect the decision-making process and results of several specific decision trees, and the fault diagnosis results obtained generally remain accurate. Therefore, RF has strong robustness to a small amount of missing data caused by machine failure or bad operating conditions. When a large number of data are missing, it is common to fill the dataset with default or mean values. However, this processing method may change the distribution of data, introduce system bias, and thus reduce the classification accuracy of the RF algorithm.

Therefore, in order to make the most of the information available, to alleviate the adverse effect of the common filling methods on the RF when a large number of data go missing, this paper utilizes the decision path information to improve the accuracy of RF on the sample containing missing data.

According to the discussion of the decision path above, when sample x lacks several features, which are in the decision path corresponding to sample x in decision tree t, the classification results predicted by the decision tree are considered unreliable, that is, the missing feature nodes included in the decision path will reduce the credibility of the corresponding prediction results. To describe the adverse influence, the reliability score (RS) for the prediction results of each decision tree for a specific sample is defined. As is shown in the concept of local importance, the higher the local importance of a node containing missing data, the greater the adverse effect on the result. Since missing data occurs randomly, a decision path may consist of multiple split nodes that contain the same or different missing data, and the classification bias gets larger as the number of problem nodes increases. The sum of the local importance of all problem nodes in a decision path can indicate the degree of data loss that is unfavorable to the classification result. Considering that the length of the decision path of different samples in different decision trees is not the same, in order to compare the relative weakening effect on the classification accuracy, the ratio of the sum of the local importance of the reliable nodes to that of all nodes in the decision path can be calculated in the case of missing data. The reliability score of decision tree t can be denoted with respect to sample x as follows:

(6) $$RS_{t}\left(x,y\right)=1-\frac{\sum_{k^{\prime }\in {𝒟𝒫}_{\mathrm{miss}}(t,x)}LI_{k^{\prime }}^{t}(y)}{\sum_{k\in 𝒟𝒫(t,x)}LI_{k}^{t}(y)}$$

where 𝒟𝒫(t,x) and $𝒟{𝒫}_{\mathrm{miss}}(t,x)$ are a collection of all the nodes of the decision path in the decision tree t, and a collection of all the corresponding missing nodes, respectively.

**Figure 2:**
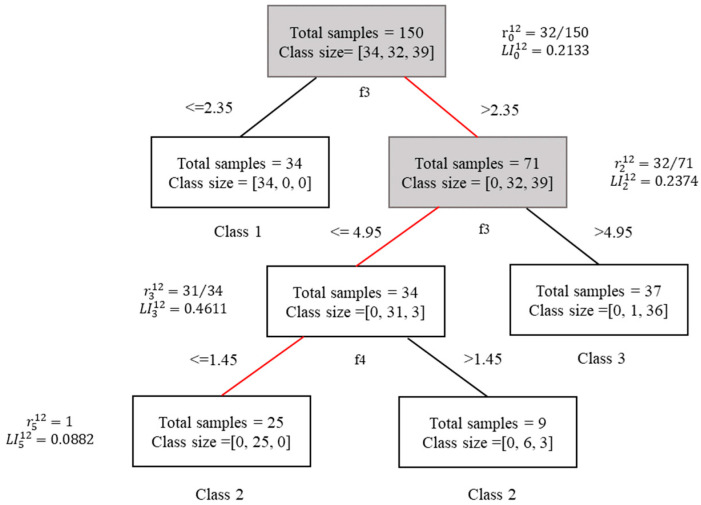
The process of classifying iris dataset with missing data.

The iris data are still used as an example to illustrate how RS is calculated. By analysis in Section 2.1, the test sample $x_{6}$ in the 12th decision tree passes through $n_{0}\to n_{2}\to n_{3}\to n_{5}$, and the predicted result is 2. If $f_{3}$ is missing in $x_{6}$, then the nodes $n_{0}$ and $n_{2}$ containing the missing feature $f_{3}$ are unreliable, as shown in Figure 2.

According to the definition, the RS value of the 12th decision tree for sample $x_{6}$ is as follows:

(7) $$RS_{12}\left(x=x_{6},y=2\right)=\frac{\left(0.2133+0.2374+0.4611+0.0882\right)-\left(0.2133+0.2374\right)}{\left(0.2133+0.2374+0.4611+0.0882\right)}=0.5493$$

In the RF, the length of the decision path of the same sample x is not the same in different decision trees. In order to measure the RS of the decision trees with different decision path lengths, all the RS in the RF are normalized in this paper as follows:

(8) $$RS_{t}^{\mathrm{norm}}\left(x,y\right)=\frac{RS_{t}\left(x,y\right)-RS_{\min}\left(x,y\right)}{RS_{\max}\left(x,y\right)-RS_{\min}\left(x,y\right)}$$

where $RS_{\min}\left(x,y\right)$ and $RS_{\max}\left(x,y\right)$ represent the minimum and maximum values of RS values for all decision trees in a random forest, respectively, as follows:

(9) $$RS_{\min}\left(x,y\right)=\underset{t^{\prime }\in \left\{1,\ldots T\right\}}{\min}\;RS_{t^{\prime }}\left(x,y\right)$$

(10) $$RS_{\max}\left(x,y\right)=\underset{t^{\prime }\in \left\{1,\ldots T\right\}}{\max}\;RS_{t^{\prime }}\left(x,y\right)$$

For cases with missing data, this paper retains the original prediction results of each classifier because although some data in the test samples go missing, remaining data are still valid and retain important information. Besides, this paper uses reliability scores to revise the prediction results according to missing data contained in the decision path. The specific amendment process will be discussed according to two different voting rules.

When applying hard voting, the test sample x is inputted, and each decision tree can obtain the predicted label value y and the corresponding reliability scores, denoted as $RS_{t}^{\mathrm{norm}}\left(x,y\right)$. For different classifiers with the same prediction label, the sum of their reliability scores is used to obtain the total reliability predicted in the category. The label with the highest total reliability scores in all categories is taken as the final prediction result.

(11) $$H_{DPRF,hard}\left(x\right)=\underset{y\in Y}{argmax}\;\left\{\overset{T}{\underset{t=1}{\sum }}v_{t}\left(x,y\right)\cdot RS_{t}^{norm}\left(x,y\right)\right\}$$

When applying soft voting, the test sample x is inputted, and each decision tree outputs the predicted probability of each category and the reliability scores of the classifier under the condition of missing data. The prediction probability of each category is multiplied by the reliability scores to obtain the revised predicted probability of each category, and the revised prediction probabilities of all sub-classifiers in the RF are added to obtain the total prediction of each category probability. The category with the highest predicted probability is taken as the final classification result of the sample x.

(12) $$H_{DPRF,hard}\left(x\right)=\underset{y\in Y}{argmax}\;\left\{\frac{1}{T}\overset{T}{\underset{t=1}{\sum }}P_{t}\left(x,y\right)\cdot RS_{t}^{norm}\left(x,y\right)\right\}$$

The soft voting rule summarizes the probabilities of all categories in all decision trees before voting, while the hard voting rule first votes in each decision tree and then aggregates the results. The former rule can better retain the classification probability information of each decision tree to the final result, while the latter one first votes in the decision tree, losing the probability information too early. Therefore, the diagnosis accuracy is theoretically higher under the soft voting rule.

By repeating the above procedures, the classification results for all the samples can be obtained.

The proposed DPRF algorithm is summarized as follows:

Stage 1: data collection and model training

(1). Collecting data: collect datasets for X and Y: $X_{N\times D}=\left\{\left(x_{1,1},\ldots ,x_{1,D}\right),\ldots ,\;\left(x_{N,1},\ldots ,x_{N,D}\right)\right\}$ and $Y_{N\times 1}=\left\{y_{1},\ldots ,y_{N}\right\}$;
(2). Generating training and testing sets: Split the datasets of X and Y into training and testing sets: $\left(X_{train},Y_{train}\right)$ and $\left(X_{test},Y_{test}\right)$, of which the sample sizes correspond to $N_{train}$ and $N_{test}$, respectively. Note that $X_{train}$ and $Y_{train}$ do not contain any missing data;
(3). Training RF model: train the RF with complete data $\left(X_{train},Y_{train}\right)$;
(4). Obtaining nodes’ LI scores: calculate the LI scores for all the nodes on the trees in the RF according to the above Equation (1).

Stage 2: classification with incomplete data

(1). Identifying decision paths for test sample: consider a test sample $x_{test}$ in $X_{test}$ with incomplete data, for each tree $f_{t}$ in the RF, identify the corresponding decision path and decision nodes set by 𝒟𝒫(t,x) and $𝒟{𝒫}_{\mathrm{miss}}(t,x)$;
(2). Calculating reliability scores for decision trees: for all the trees in the RF, calculate their normalized RS scores according to Equations (6) and (8);
(3). Obtaining final classification result for $x_{test}$: calculate the final weighted result according to the hard or soft voting procedures in Equations (11) and (12).

In short, the DPRF model firstly uses the complete industrial process dataset to train the RF model. Then, for the actual process samples that may contain missing data, DPRF automatically determines the location of the missing features of the sample, and completes the calculation of the decision paths, reliability scores and final FDD results on all decision trees. DPRF uses the information retained in the dataset to correct the judgment of RF, and alleviates the influence of subjective factors.

## 3. Case Study of the Tennessee Eastman Process

This section tests DPRF, together with other classic FDD algorithms in the famous Tennessee Eastman process, to examine the performance of DPRF and compare it with other methods [43]. At the same time, other fault diagnosis methods, such as the back propagation neural network (BP), deep belief neural network (DBN), and radial basis function (RBF) neural network, are also used to compare diagnostic performance. In this paper, we use overall accuracy (OACC) [44,45] and label-based accuracy (LACC) [45,46] to evaluate the performance of each classification algorithm in detecting and correctly classifying faults. The expressions of OACC and LACC are as follows:

(13) $$LACC_{i}=\frac{TP_{i}+TN_{i}}{TP_{i}+TN_{i}+FP_{i}+FN_{i}}$$

(14) $$OACC=\frac{1}{\left|Y\right|}\overset{\left|Y\right|}{\underset{y=1}{\sum }}LACC_{y}$$

where |Y| is the number of elements in collection Y, that is, the total number of labels. $TP_{i}\;$ is the number of data that are predicted as fault i, which is actual fault i, while $TN_{i}$ is the number of data that are predicted as fault i, which is not fault i, indeed. $FP_{i}$ is the number of data that are predicted as normal or other faults, which is actual fault i, while $FN_{i}$ is the number of data that are predicted as normal or other faults, which is not that case, indeed. 

It can be observed that LACC represents the classification accuracy rate on each classification label, while OACC represents the average classification accuracy rate on all the labels.

### 3.1. Tennessee Eastman Process Description

**Figure 3:**
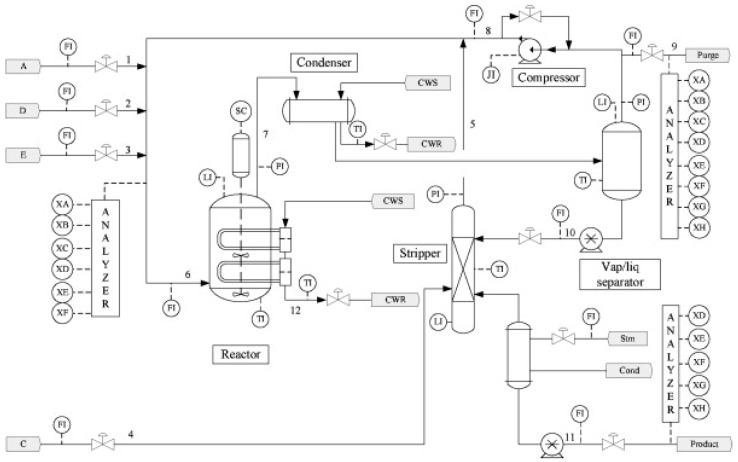
Process flowsheet of TE process. Reprinted with permission from ref. [52]. Copyright 2012 Elsevier.

The Tennessee Eastman (TE) benchmark chemical process was first introduced by Downs and Vogel in 1993 [47], to evaluate the performance of all kinds of process monitoring and fault diagnosis methods [48,49,50]. The TE process is constructed with the following five major operation units: a reactor, a product condenser, a vapor–liquid separator, a recycle compressor, and a product stripper. A total of 11 manipulated variables and 41 measured variables are contained in the TE process, and the data can be attributed to 21 fault types. These 21 fault types are detailed in Table 2. The TE process is shown in the flowchart in Figure 3.

### 3.2. Results

The training set of the standard dataset for the TE process (http://web.mit.edu/braatzgroup/links.html, accessed on 14 September 2021) contains 500 samples that are generated from the normal state, and 480 samples that are generated from each fault state. For the testing set, there are 960 samples in the normal state and each fault state. For the testing set of each fault type, the fault is introduced at the 160th sample. Thus, the first 160 samples are in the normal state and the last 800 samples are in the corresponding fault state [53]. Because many FDD algorithms have been tested using a standard dataset by other researchers, it is convenient to compare the results to the general methods by experimenting on the same standard dataset. 

In recent fault diagnosis studies, methods based on neural networks and deep learning have emerged [54,55,56,57]. BP, which was developed in 1986, by scientists led by Rumelhart and McClelland, is a multi-layer feed-forward neural network that is trained by error reverse propagation algorithms and is the most widely used neural network. DBN consists of several restricted Boltzmann machine (RBM) layers and a BP layer, and its unsupervised pre-training can extract high-level abstract representations from the input data [58,59,60]. RBF is often used to approximate multi-dimensional surfaces by a linear combination of radial symmetric functions, based on Euclidean distance. In recent years, RBF has been used in FDD [61,62,63,64]. This section compares the proposed DPRF with these methods.

Some previous research on missing data modelling created loss based on the following three missing patterns: missing completely at random (MCAR), missing at random (MAR), and missing not at random (MNAR) [65,66]. However, it is sometimes difficult to distinguish the missing mechanisms. This paper proposes a random data loss construction method in the entire test set, randomly setting missing positions on the test dataset matrix $D_{16,800\times 52}$ at the missing rates mr=0.1,0.2,0.3,0.4, with corresponding amounts of missing data 1680, 3360, 5040, and 6720, respectively. This method does not rely on the three common patterns of missing data. It has better compatibility in various situations. Such a setting also makes the position and number of missing features differ among different samples, making it easier to examine different FDD algorithms under various missing situations. Besides, the amount of missing data in this setting is larger, in which case many FDD algorithms tend to fail. From the discussion in the previous section, DPRF can flexibly search for the missing locations sample by sample, automatically calculate the decision paths and corresponding missing nodes of each sample on all decision trees, and then obtain the RS of each decision tree based on the importance of the missing nodes. Therefore, DPRF can automatically adapt to situations where different locations and different numbers of sample data go missing, without manual intervention. DPRF has better universality compared with other FDD algorithms. 

**Figure 4:**
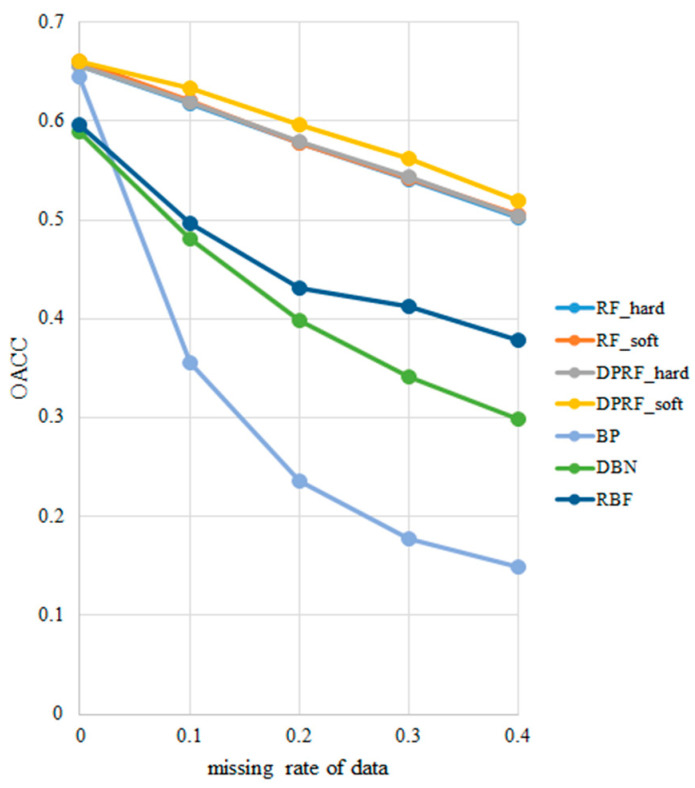
Comparison of OACC values at different missing rates.

The OACC values of BP, DBN, RBF, the traditional RF algorithm, and its improved version DPRF at each missing rate are plotted in Figure 4, and the details are listed in Table 3. When the test set is complete, the RS of all the decision trees in RF and DPRF are equal to one, that is, there is no need to modify the prediction results. In this case, the OACC and LACC of DPRF are the same as traditional RF, and both algorithms perform well, with OACC values as high as 66.07% under the soft voting rule and 65.64% under the hard voting rule. From the experimental results, the OACC value obtained by the soft voting rule is higher than that obtained by the hard voting rule at the same missing data rate in either RF or DPRF. This experimental result is consistent with the conclusion in the theoretical analysis.

DPFR based on hard voting, and RF based on both hard and soft voting have approximately the same test results at missing rates of 0.1, 0.2, 0.3, and 0.4 in the standard dataset. The curves of the three algorithms roughly coincide. However, it can be observed from Table 3 that DPRF is better than RF under the hard voting rule. In contrast, the DPRF algorithm based on the soft voting rule is significantly better than other algorithms, with OACC values that are 1.4%, 1.8%, 1.94%, and 1.47%, respectively, higher than that of the RF algorithm under the same rule. The OACC values of BP, DBN, and RBF fluctuate significantly when missing data occurs.

## 4. Discussion

In order to compare the stability of each algorithm when data are lost, the robustness evaluation function is defined as follows:

(15) $$Robustness_{i,mr}=\{\begin{array}{c} 4,\;if\;\left|ratio_{i,mr}\right|\leq 0.05\\ \begin{array}{c} 3,\;if\;0.05<\left|ratio_{i,mr}\right|\leq 0.1\\ 2,if\;0.1<\left|ratio_{i,mr}\right|\leq 0.2\\ 1,if\;\left|ratio_{i,mr}\right|>0.2 \end{array} \end{array}$$

(16) $$Robustness_{i,mr}=\frac{OACC_{i,mr}-OACC_{i,mr-0.1}}{OACC_{i,mr}}$$

$$i\epsilon \left\{{\mathrm{RF}}_{\mathrm{hard}},{\mathrm{RF}}_{\mathrm{soft}},{\mathrm{DPRF}}_{\mathrm{hard}},{\mathrm{DPRF}}_{\mathrm{soft}},\mathrm{BP},\mathrm{DBN},\mathrm{RBF}\right\},mr=0.1,0.2,0.3,0.4$$

where $OACC_{i,mr}$ represents the overall accuracy rate of algorithm i at the missing rate mr. $ratio_{i,mr}$ represents the relative change rate of OACC for algorithm i, between the missing rate mr and the previous missing rate (mr−0.1). When the missing rate gradually increases, the relative change rate of the OACC of an algorithm increases, indicating that the robustness of the algorithm is poor at this stage, as shown by the lower robustness score obtained. Otherwise, the smaller the relative change rate of the overall accuracy rate, the better the robustness of the algorithm at this stage, thus the higher the robustness score obtained. Therefore, the robustness scores are set, ranging from 1 to 5, as shown in the above formula. 

Based on the function, we evaluate the stability of all seven FDD algorithms in this paper, for samples containing missing data. The results are listed in Table 4.

As can be observed from Table 4, DRPF based on soft voting rules has the best robustness and the strongest adaptability to data loss, while BP is the most sensitive to data loss. The average robustness scores of DPRF based on the hard voting rule is three, which is the same as that of RF based on both soft and hard voting rules. These algorithms have good stability for data loss. RBF’s average robustness score is up to 2.75, and its performance is the best among the three neural network models, but is inferior to that of the DRPF and RF models. This feature is qualitatively revealed in Figure 4, where BP’s OACC curve is the steepest, with the lowest robustness score, while the OACC curves of RF and DPRF are relatively flat, with the robustness scores ranking high.

**Figure 5:**
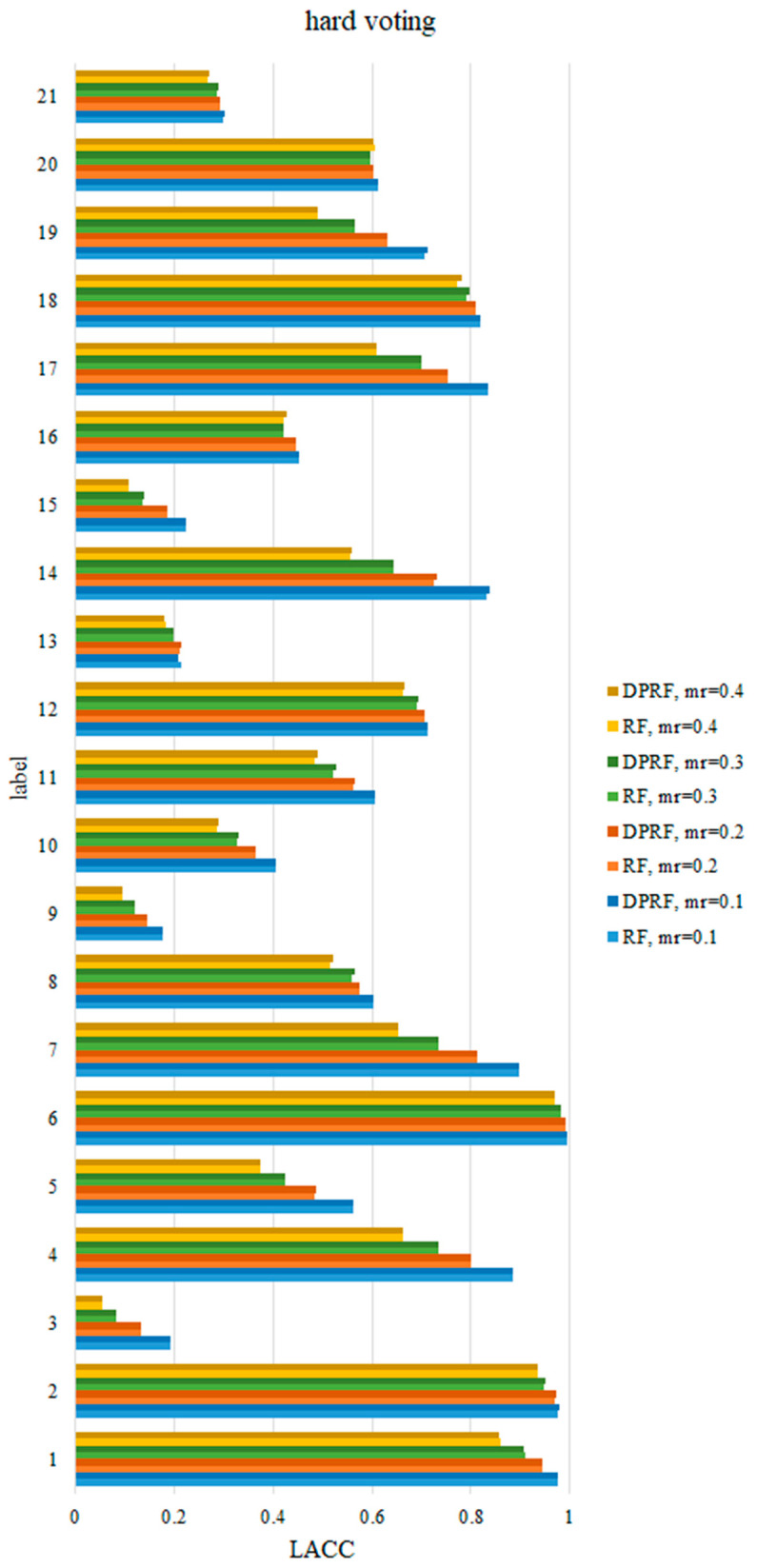
Comparison of LACC of RF and DPRF at different missing rates based on hard voting.

**Figure 6:**
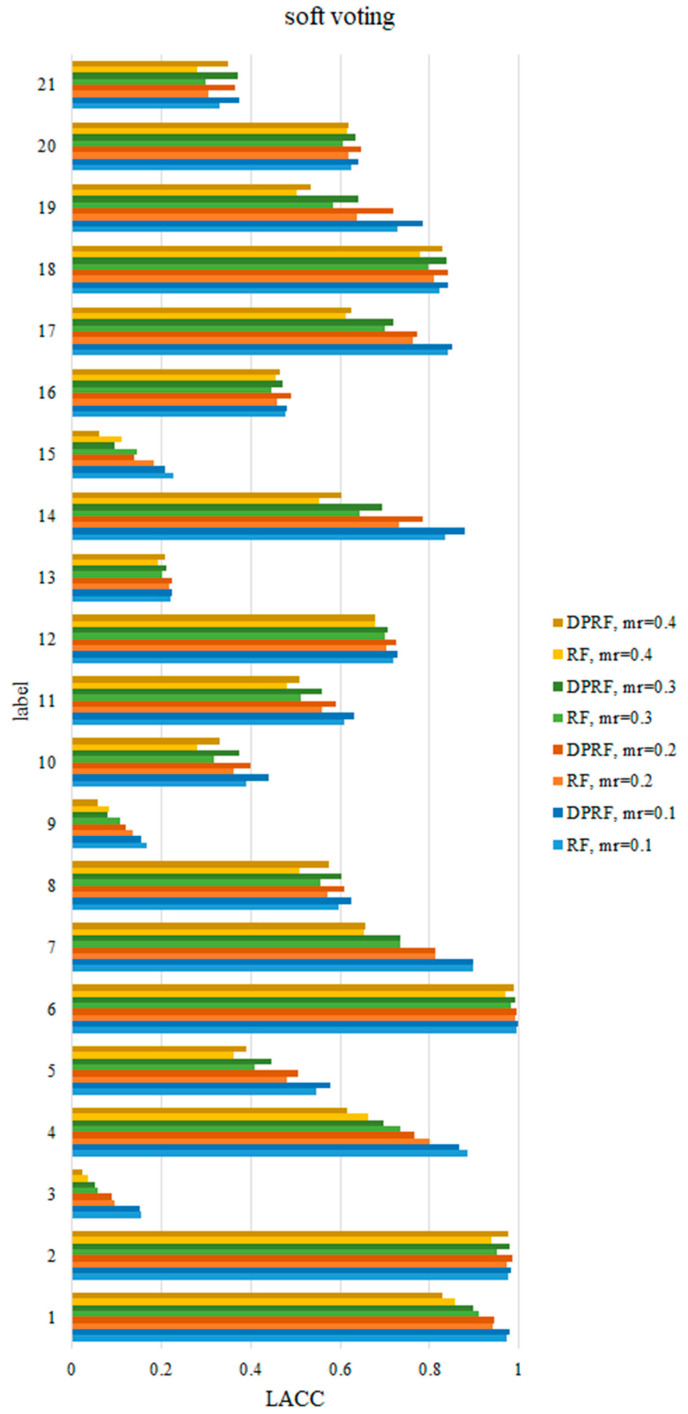
Comparison of LACC of RF and DPRF at different missing rates based on soft voting.

As mentioned above, OACC represents the average classification accuracy rate on all labels. In order to further compare the detection performance of the FDD algorithms on various fault labels, it is necessary to compare the LACC values of each fault. Since RF and DPRF both perform well in terms of OACC, this section only compares the LACC values of the two algorithms on various faults. Table 5 shows the specific results of LACC at the missing rate 0.1, under the soft voting rule. The other results are plotted in Figure 5 and Figure 6. Figure 5 shows that for the hard voting rule, the diagnostic accuracy rate of DPRF in each category is generally higher than that of RF at the same missing rate. As the missing rate increases, the accuracy of DPRF and RF in each category decreases, but DPRF still has an advantage in diagnostic accuracy compared to RF. Similarly, under the soft voting rule in Figure 6, the LACC of DPRF is improved in most categories, compared to that of RF. Moreover, in Figure 5, it is clear that the sensitivity of each label to the missing data is different. As the missing rate increases for some categories, such as 7, 14, and 17, the accuracy rate sharply decreases, while for other categories, such as 6, 16, and 20, the accuracy rate only fluctuates slightly.

In order to quantify the comparison of the LACC of each category on the two algorithms more clearly, Table 6 lists the summary of the LACC of RF and DPRF under soft voting rules at different missing rates. Taking the first line of the table as an example, at the missing rate 0.1, there are 16 categories where the LACC value of DPRF is greater than or equal to that of RF among 21 fault categories, and the LACC value of DPRF is less than that of RF in the remaining 5 categories.

**Figure 7:**
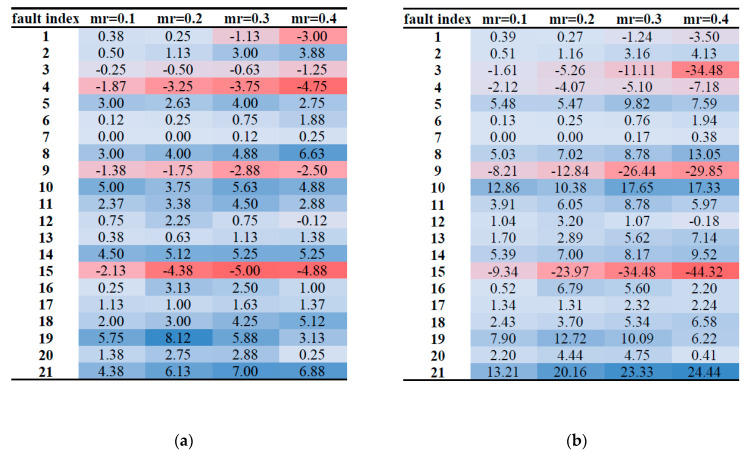
Details of LACC improvement by DRPF under the soft voting rule. (**a**) Absolute change value of LACC, (**b**) relative change value of LACC (%).

It can be concluded from Table 6 that for about 70% of the categories under the soft voting rule, the LACC of DPRF is higher than that of RF, which indicates that the proposed DPRF algorithm improves the diagnosis effects of most of the fault categories, and, finally, promotes the OACC. It means that DPRF has better adaptability to different types of fault diagnoses. Figure 7 shows the LACC improvement of 21 types of faults using DPRF. Figure 7a depicts the absolute change value of LACC, while Figure 7b depicts the relative change value of LACC. When the LACC value is not improved by DPRF, the calculation result is a negative number filled with red. The more negative the result, the darker the red. Otherwise, the calculation result is a positive number filled with blue. The more positive the result, the darker the blue.

Most of the areas in Figure 7 are blue. However, the diagnosis effect of the 3, 4, 9, and 15 types of faults have not been improved under all the missing rates calculated by DPRF, and when the missing rates reach 0.3 and 0.4, the diagnostic effect of the first type of faults is also unable to be improved, despite the introduction of the new algorithm. Previous studies [46,67] have concluded that the detection of faults 3, 9, and 15 is very difficult, as there are not any observable changes in the means, variance, or the peak time. Therefore, these three faults cannot always be detected by any statistical technique. Random forest is a statistical algorithm based on information entropy, so its low accuracy is reasonable in the diagnosis of these three types of faults, and it is difficult for DPRF to improve the situation. Chiang et al. [68] proposed that the fourth type of fault will only cause the mean and standard deviation of each variable to differ less than 2% between the faulty status and normal operation. This phenomenon makes the observed signal belonging to fault four similar to the signal under the normal state, resulting in the distinction of the fourth type of fault difficult. Therefore, it is difficult to improve the diagnostic accuracy of the fourth type of fault [69]. It is detected that fault one fails to be improved by DPRF if the missing rate is over 0.3; however, the real reason behind the phenomenon is not clear. Further studies should be carried out.

In conclusion, the test on the TE process confirms the DPRF’s effectiveness in accuracy and stability in FDD. DPRF can be easily applied to other chemical processes of different amounts of samples and features, and it will outperform other traditional FDD algorithms in cases of incomplete data. This is not surprising, since the proposed method utilizes the correction procedure, which helps to alleviate the adverse impacts of missing data. DPRF is also adaptable to harsh working conditions where large amounts of data are hard to detect, and thus numerous vacancies occur in records, since the experiment shows that DPRF’s diagnostic accuracy maintains a high value at the missing rate 0.4. This is definitely useful in the fault diagnosis of extreme cases, such as large-scope instrument failure.

## 5. Conclusions

This paper proposes an improved RF algorithm based on the decision path named DPRF. Firstly, an ensemble classifier with a fixed internal tree structure is constructed on the basis of the traditional RF algorithm. Next, for each test sample input to RF, the classifier returns a specific decision path, which contains features for splitting to reach the final classification result. Then, according to the prediction results, the local importance for the nodes and reliability scores is calculated, and the original prediction results are revised. Finally, two different voting rules are used for obtaining the final classification results.

In addition to the DPRF algorithm under the two voting rules, five other FDD strategies are applied, including the RF algorithm based on the hard voting rule, the RF algorithm based on the soft voting rule, BP, DBN, and RBF, to show the effectiveness of the DPRF algorithm. Compared with other methods, DPRF shows strong stability when the dataset is incomplete. When the data missing rate is set from 0.1 to 0.4, DPRF always performs better than the traditional RF, especially under the soft voting rule, with the OACC value increasing by more than 1%. The diagnostic accuracy rate of DPRF is far ahead of that of BP, DBN, and RBF. With the increase in the missing rate, the proposed DPRF algorithm also maintains a high robustness score, and its average robustness score, reaching 3.25, is the best among the compared algorithms. Additionally, DPRF has excellent performance in terms of the diagnostic accuracy rate of local categories, when comparing RF and DPRF under the same voting rule and missing rate. Therefore, DPRF has improved the overall accuracy, robustness, and label accuracy compared to the traditional RF algorithm. When applied in actual chemical processes, where missing data are ubiquitous, DPRF has advantages in accurately detecting and diagnosing faults, and maintains its applicability in the case of large amounts of missing data. 

The correction coefficient is a common approximation strategy that is applied in the engineering field. This article draws on this idea to introduce the reliability coefficient, consequently improving the diagnostic accuracy. In the future, when encountering other abnormal data, except for missing data, other existing machine learning algorithms can also be modified with the idea of correction. However, it should be mentioned that the proposed DPRF relies on the choice of modified strategy, i.e., different definitions of correction coefficients will generate different results. Besides, the computational time of DPRF is longer compared to the other four FDD algorithms, due to the embedding of the iterative correction procedure. These shortcomings will limit the practical application of this method in complex chemical processes. In future studies, we will discuss how to combine DPRF with feature extraction strategies to reduce the computation cost, which will help to effectively diagnose in industrial chemical processes.

## Figures and Tables

**Table 1:** The feature values of test sample $x_{6}$ in the iris dataset.

	$f_{1}$	$f_{2}$	$f_{3}$	$f_{4}$
$x_{6}$	5.6	2.5	3.9	1.1

**Table 2:** Process faults for Tennessee Eastman process. Reprinted with permission from ref. [51]. Copyright 2019 Elsevier.

Fault	Process Variable	Type
IDV (1)	A/C feed ratio, B composition constant (stream 4)	Step
IDV (2)	B composition, A/C feed ratio constant (stream 4)	Step
IDV (3)	D feed temperature (stream 2)	Step
IDV (4)	Reactor cooling water inlet temperature	Step
IDV (5)	Condenser cooling water inlet temperature	Step
IDV (6)	A feed loss (stream 1)	Step
IDV (7)	C header pressure loss-reduced availability (stream 4)	Step
IDV (8)	A, B, and C feed compositions (stream 4)	Random variation
IDV (9)	D feed temperature (stream 2)	Random variation
IDV (10)	C feed temperature (stream 4)	Random variation
IDV (11)	Reactor cooling water inlet temperature	Random variation
IDV (12)	Condenser cooling water inlet temperature	Random variation
IDV (13)	Reaction kinetics	Slow shift
IDV (14)	Reactor cooling water valve	Sticking
IDV (15)	Condenser cooling water valve	Sticking
IDV (16)	Unknown	Unknown
IDV (17)	Unknown	Unknown
IDV (18)	Unknown	Unknown
IDV (19)	Unknown	Unknown
IDV (20)	Unknown	Unknown
IDV (21)	Valve position constant (stream 4)	Constant position

**Table 3:** OACC values of the seven FDD algorithms.

Missing Rate	$RF_{hard}$	$RF_{soft}$	$DPRF_{hard}$	$DPRF_{soft}$	BP	DBN	RBF
0	0.6564	0.6607	0.6564	0.6607	0.6452	0.5892	0.5967
0.1	0.6179	0.6201	0.6187	0.6340	0.3561	0.4809	0.4964
0.2	0.5784	0.5783	0.5791	0.5963	0.2361	0.3986	0.4308
0.3	0.5411	0.5422	0.5429	0.5616	0.1777	0.3410	0.4130
0.4	0.5027	0.5052	0.5043	0.5199	0.1485	0.2981	0.3780

**Table 4:** Robustness scores for the seven algorithms.

Missing Rate	$RF_{hard}$	$RF_{soft}$	$DPRF_{hard}$	$DPRF_{soft}$	BP	DBN	RBF
0.1	3	3	3	4	1	2	2
0.2	3	3	3	3	1	2	2
0.3	3	3	3	3	1	2	4
0.4	3	3	3	3	2	2	3
Average	3	3	3	3.25	1.25	2	2.75

**Table 5:** LACC values of RF and DPRF based on soft voting rules (missing rate = 0.1).

Fault Type	$RF_{soft}$	$DPRF_{soft}$
1	0.9738	0.9775
2	0.9763	0.9813
3	0.1550	0.1525
4	0.8863	0.8675
5	0.5475	0.5775
6	0.9950	0.9963
7	0.8988	0.8988
8	0.5963	0.6263
9	0.1675	0.1538
10	0.3888	0.4388
11	0.6075	0.6313
12	0.7200	0.7275
13	0.2200	0.2238
14	0.8350	0.8800
15	0.2275	0.2063
16	0.4788	0.4813
17	0.8400	0.8513
18	0.8225	0.8425
19	0.7275	0.7850
20	0.6263	0.6400
21	0.3313	0.3750

**Table 6:** Summary of LACC of RF and DPRF based on soft voting rules.

Missing Rate	$RF_{soft}$	$DPRF_{soft}$
0.1	5	15
0.2	4	17
0.3	5	16
0.4	6	15

## Data Availability

Data available on request due to restrictions, e.g., privacy or ethical.

## Nomenclatures

T	the total number of decision trees in the random forest
Y	a collection of all target labels y
t	decision tree t
x	single sample
y	the predicted label of x in the random forest, y∈Y
$r_{k}^{t}\left(y\right)$	the ratio of samples belonging to label y on node k in decision tree t
$LI_{k}^{t}\left(y\right)$	the local importance of node k in decision tree t with predicted label y
$f_{t}\left(x\right)$	the prediction of sample x in decision tree t
$v_{t}\left(x,y\right)$	the binary voting variable of decision tree t on whether the sample prediction equals to y
$P_{t}\left(x,y\right)$	the possibility that the sample prediction equals to y in decision tree t
$RS_{k}\left(x,y\right)$	the reliability score calculated by the decision path sample x passes in decision tree t
$RS_{min}\left(x,y\right)$, $RS_{max}\left(x,y\right)$	the minimum and maximum values of RS in all decision trees
$H_{RF,hard}$, $H_{RF,soft}$, $H_{DPRF,hard}$, $H_{DPRF,soft}$	the voting results of RF and DPRF under the hard and soft voting rule, respectively
𝒟𝒫(t,x),$𝒟{𝒫}_{\mathrm{miss}}(t,x)$	the collection of all the nodes of the decision path in decision tree t, and the collection of all the corresponding missing nodes, respectively.
